# An Intelligent Waste-Sorting and Recycling Device Based on Improved EfficientNet

**DOI:** 10.3390/ijerph192315987

**Published:** 2022-11-30

**Authors:** Zhicheng Feng, Jie Yang, Lifang Chen, Zhichao Chen, Linhong Li

**Affiliations:** 1 Department of Electrical Engineering and Automation, Jiangxi University of Science and Technology, Ganzhou 341000, China; 2 Jiangxi Provincial Key Laboratory of Maglev Technology, Ganzhou 341000, China; 3 Ganjiang Innovation Academy, Chinese Academy of Sciences, Ganzhou 341000, China; 4 Department of Science, Jiangxi University of Science and Technology, Ganzhou 341000, China

**Keywords:** waste sorting and recycling, image classification, artificial intelligence, sustainable development, EfficientNet

## Abstract

The main source of urban waste is the daily life activities of residents, and the waste sorting of residents’ waste is important for promoting economic recycling, reducing labor costs, and protecting the environment. However, most residents are unable to make accurate judgments about the categories of household waste, which severely limits the efficiency of waste sorting. We have designed an intelligent waste bin that enables automatic waste sorting and recycling, avoiding the extensive knowledge required for waste sorting. To ensure that the waste-classification model is high accuracy and works in real time, GECM-EfficientNet is proposed based on EfficientNet by streamlining the mobile inverted bottleneck convolution (MBConv) module, introducing the efficient channel attention (ECA) module and coordinate attention (CA) module, and transfer learning. The accuracy of GECM-EfficientNet reaches 94.54% and 94.23% on the self-built household waste dataset and TrashNet dataset, with parameters of only 1.23 M. The time of one recognition on the intelligent waste bin is only 146 ms, which satisfies the real-time classification requirement. Our method improves the computational efficiency of the waste-classification model and simplifies the hardware requirements, which contributes to the residents’ waste classification based on intelligent devices.

## 1. Introduction

In recent years, as urbanization and living standards have increased, the variety and quantity of waste has increased dramatically [1], putting enormous pressure on resource use, environmental safety, and economical recycling. Urban residents are the main producers of household waste, and they participate in household waste sorting. Recycling is an effective way to utilize waste resources, reduce the quantity of waste, and contribute to sustainable development [2]. However, due to many different categories of waste, extensive sorting knowledge is required. It is difficult to translate residents’ willingness for waste sorting into actual action. As a result, many countries have started researching intelligent waste sorting and recycling devices, which were applied in engineering practice [3,4]. Intelligent recognition of waste categories is a prerequisite for sorting and recycling. Computer vision technology and deep learning technology can automatically detect and classify waste categories [5,6], providing technical support for waste sorting and recycling.

The convolutional neural network (CNN) is one of the main branches of deep learning, and it is the mainstream image-recognition method nowadays. With the rapid development of deep learning technology, CNN has made significant achievements in image classification [7]. Numerous researchers have used the CNN to solve waste image classification tasks [8,9], and have achieved a series of achievements. Ref. [10] improved ResNet18 with a self-monitoring module to enhance the feature map representation, achieving 95.87% accuracy on the TrashNet dataset. Ref. [11] optimized DenseNet121 with data augmentation and genetic algorithms, achieving 99.6% accuracy on the TrashNet dataset. Ref. [12] implemented waste image classification by ResNet50, achieving 95.3% accuracy on the self-built dataset. It is feasible to achieve accurate and reliable waste classification by CNN. However, the above research articles use the large CNN model, which performs well on accuracy. However, they have many parameters, and the inference of the models requires many floating point operations [13,14]. It is impractical to have high-performance chips in every waste-sorting device, which makes their application difficult. Therefore, aiming at embedded devices or platforms with limited resources, numerous researchers have started to explore lightweight CNN models for waste image classification, which are light, efficient, and have acceptable performance. Ref. [15] implemented waste image classification by MobileNetv3 and achieved 94.26% accuracy on the self-built dataset, with a single infer time of only 261.7 ms. Ref. [16] proposed the GCNet based on ShuffleNetv2, which was improved by parallel space and channel attention module, FRelu, and transfer learning, the model parameters were 1.3 M, and the single infer time on the Raspberry Pi 4B was only 105 ms, with an accuracy of 97.9% on the self-built dataset.

The scholars mentioned above have researched waste image classification and verified the effectiveness of CNN, which has practical implications for automatic waste classification and recycling. However, most of these research efforts are based on large models to implement waste image classification, with less focus on lightweight and actual applications, which is not conducive to them being used in real life. Therefore, the aim and novelty of this paper is the proposition of a lightweight and efficient model for waste image classification, which is applied to the intelligent waste bin that we designed. Our proposed method can automatically sort and collect the input household waste, including recyclable waste, hazardous waste, kitchen waste, and other waste, which provides a feasible solution to residents for waste sorting.

Overall, the main contributions of this work include the following.

(1) An intelligent waste bin has been designed, which can automatically collect the waste put in, improve the efficiency of waste sorting by residents, and reduce the separation work of collection facilities.
(2) We propose an improved EfficientNet, named GECM-EfficientNet, which accurately classifies different categories of waste by fewer parameters.
(3) We use transfer learning [17] to initialize the model parameters during training, optimizing the performance of the model without adding extra computation.
(4) Our waste-classification model balances speed and accuracy with good real-time performance on edge devices, which can reduce hardware costs.

The paper is structured as follows: Section 2 reviews the work related to the large model and lightweight model. Section 3 describes the working process of the intelligent waste bin and the detailed design of the proposed model. Section 4 describes the experimental results and analysis of different datasets. Finally, Section 5 concludes the work of this paper.

## 2. Related Work

Waste-sorting and recycling devices require frequent forward inference of models, which can be computationally expensive. Unfortunately, it is impractical to equip each device with a high-performance graphics processing unit (GPU), which results in high hardware costs. Cloud deployment [18,19] can free models from reliance on local computation, but this is heavily dependent on the Internet. When there is no network or poor network connectivity, cloud deployment is not possible. Compared with large models, lightweight models tend to be slightly less accurate, but smaller and faster. Therefore, for waste sorting and recycling devices, local deployment of lightweight models would be an effective measure.

In 2012, AlexNet [20] won the ImageNet image classification competition, triggering the boom in CNN for image classification, giving rise to a series of models, such as VGG [21], GoogleNet [22], and ResNet [23], with superior performance. Ref. [24] implements three VGGs in tandem for electrocardiogram classification, achieving high accuracy of 97.23% on the PTB-XL dataset. Ref. [25] used transfer learning to improve the VGG, achieving 98.4% and 95.71% accuracy on the self-built grape and tomato pest datasets. Refs. [26,27] achieve classification of plants and botrytis by ResNet, with an accuracy of over 98%. Ref. [28] initialized GoogleNet through transfer learning, which achieved up to 99.94% accuracy on the self-built northern maize leaf blight dataset. These papers implemented image classification through large models. They excelled in accuracy, all achieving over 95% accuracy. However, large models require large memory and hardware resources, which hinders their usefulness on resource-limited embedded devices. For this, lightweight models would be a viable solution.

In 2016, ref. [29] first used lightweight ideas to design models and proposed SqueezeNet, with a model size of 0.5 M. After that, numerous developers continued to explore lightweight models, proposing MobileNet [30,31], ShuffleNet [32,33], and EfficientNet [34] (EfficientNetB0- EfficientNetB7). Among the mainstream models, EfficientNet achieves the most excellent ImageNet accuracy and has a highly efficient execution; thus, it is widely used in image classification. Ref. [35] improves EfficientNetB0 by adjusting the number of MBConv modules and using the residual structure and LeakRelu, with the parameter of 1.03 M, achieving 99.69% accuracy on the self-built human behavioral point cloud dataset. Ref. [36] initialized the weights of EfficientNetB4 by transfer learning, achieving the plant nutrient deficiency diagnosis with an accuracy of 98.52% on the DND-SB dataset. Ref. [37] embeds the spatial attention module into EfficientNetB4, which improves the accuracy by about 1% on the RFMID dataset (fundus disease dataset). Ref. [38] improves EfficientNetB0 based on the convolutional block attention module (CBAM) and coordinate attention (CA) module, improving the classification accuracy by 3.5% on the self-built cervical cancer dataset. Ref. [39] implements the printed circuit board (PCB) classification recovery model based on EfficientNetB3, improved by transfer learning, and achieves the accuracy of 94.37% on the PCB DSLR dataset.

Inspired by the many areas of EfficientNet, we chose EfficeientNet as the baseline model. Among several versions of EfficientNet, we prioritized real-time and chose EfficientNetB0, which has the fewest parameters. Based on this, we focused on exploring the application of EfficientNetB0 to waste-sorting tasks and making improvements.

## 3. Materials and Methodology

### 3.1. Waste Sorting Device

#### 3.1.1. Hardware Structure

**Figure 1:**
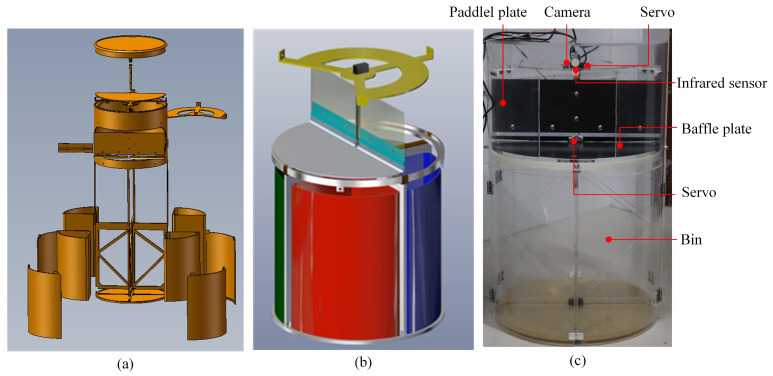
The intelligent waste bin mechanical modeling and simulation modeling are shown in (**a**) and (**b**) respectively, where they were created by SolidWorks. Subfigure (**c**) shows the physical construction of the intelligent waste bin, which is constructed by the camera, servos, bins, infrared sensor, baffle plate, and paddle plate.

In this research, we first used SolidWorks to build the mechanical structure of the intelligent waste bin. Then, the actual intelligent waste bin was built through hardware devices, Figure 1 shows its mechanical modelling, simulation modelling, and physical construction. The intelligent waste bin consists of three parts: waste recognition, actuator device, and collection device. In the waste-recognition process, the camera captures image frames, which are then passed into the classification model to identify the waste category. The actuator device consists of two servos and the attached paddle plate and baffle plate, which sort the waste into the bins by rotating at different angles. The collection device is four fan-shaped waste bins, which are set up for recyclable waste, hazardous waste, kitchen waste, and other waste according to the standards in the literature [40]. Note that the camera is HF867 with the following parameters: maximum resolution 1280 × 720; frame rate 30/s; sensitivity 39 db.

The intelligent waste bin works as follows. First, an infrared sensor senses the waste input. Then, the camera takes the image of the waste, which is passed into the waste-classification model for recognition. Finally, based on the recognition result, the servo controls the paddle plate and baffle plate to sort the waste into the corresponding bins. After the sorting is complete, the paddle plate and baffle plate return to their original position.

#### 3.1.2. Control Circuit

**Figure 2:**
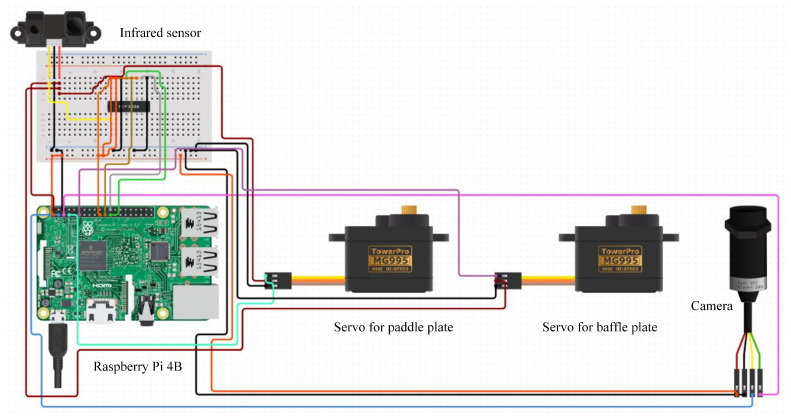
Control circuit. It is built through the Raspberry Pi 4B, camera, servos, and infrared sensor.

The Raspberry Pi 4B [41,42] is widely used in the intelligence field. We use it as the main control device in this research. The Raspberry Pi 4B processor is BROADCOM BCM2711 with 4-core CORTEX-A72, frequency is 1.5 GHZ, memory is 8 GB, and 40 expandable pins. This paper uses the Raspberry Pi 4B to deploy the waste classification model and control the infrared sensor, camera, and servo. The control circuit is shown in Figure 2.

### 3.2. Dataset Used

#### 3.2.1. Self-Built Dataset

For the task of waste classification, there is no large dedicated dataset yet. According to the literature [40] on waste classification standards, this paper establishes a scene-rich household waste dataset, and Table 1 shows the details of the dataset. Our dataset contains 18 categories of household waste, with a total of 7361 images, which are classified as recyclable waste, hazardous waste, kitchen waste, and other waste. The dataset is collected from the Internet and photography, and it is our baseline dataset. We will construct the waste-sorting device based on the dataset.

#### 3.2.2. Trashnet Dataset

This paper selects the TrashNet dataset [43] to validate the model performance. The TrashNet dataset is a small public dataset with only 2527 images, widely used in waste image classification tasks. It has six categories of waste images, namely cardboard, glass, metal, paper, plastic, and trash.

### 3.3. The Basics of Efficientnet

Typically, CNN is developed with a fixed resource budget. If resources are increased, the network can be extended to improve performance. The common method is to scale the model depth, width, and resolution. For example, by deepening the number of layers, ResNet can construct a model with 200 layers. MobileNet v2 sets the scaling factor and resolution factor, which adjusts the number of channels and the input size. Therefore, Google proposed a composite scaling method to improve the model performance, simultaneously scaling the depth, width, and input image resolution. Google designed the baseline model EfficientNetB0 and then scaled it to obtain EfficientNetB1-EfficientNetB7. On the ImageNet dataset, EfficientNet achieved contemporaneous advanced accuracy and speed.

**Figure 3:**
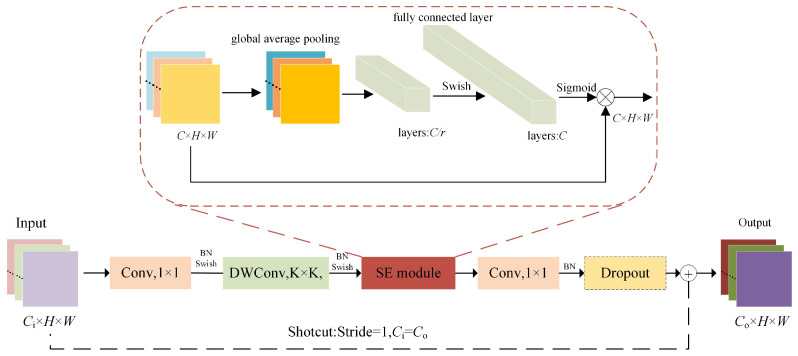
MBConv module structure.

EfficientNet adopts the mobile inverted bottleneck convolution (MBConv [31]) as the basic module, and uses the squeeze-and-excitation (SE) module [44] to calibrate the feature map by the importance of the channels. Figure 3 shows the structure of MBConv. $C_{i}$ and $C_{o}$ are input channel and output channel, respectively. *H* and *W* are height and width of the feature map, respectively. DWConv is a deepwise convolution, the kernel size of which is K. BN denotes batch normalization. Swish and Sigmoid are the activation function. In the SE module, *C* represents the channel of the feature map, and *r* is the parameter used for dimensionality reduction, which is set to 4. First, the input channels are augmented by 1×1 convolution (pointwise convolution, PW), then achieved feature extraction by 3×3 or 5×5 deepwise convolution. In the SE module, the global features are extracted through global average pooling, and then channel weights are obtained with the two fully connected layers and sigmoid. The channel weights and the feature map are multiplied channel by channel, which implements the channel weighting operation. Finally, the channels of the feature map are adjusted by PW. Shortcut and dropout are used only when the input channel and output channel are equal, and the stride is 1.

### 3.4. Improved Efficientnet

**Figure 4:**
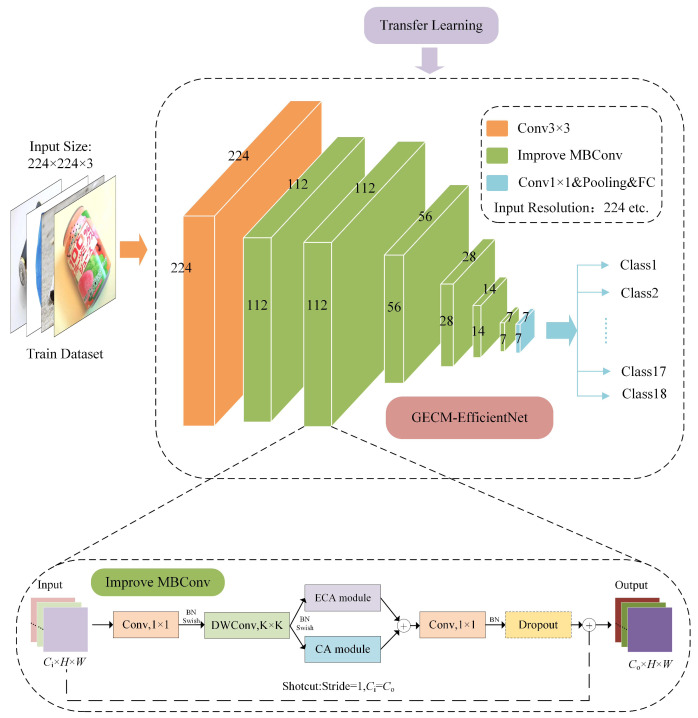
Improved EfficientNet structure.

The intelligent waste bin is used daily, requiring high accuracy and real-time waste classification models. EfficientNetB0 is a simple and elegant model, which combines the advantages of MobileNetv2, but with more efficient feature-extraction capabilities. This paper designed a lightweight and efficient waste image classification model based on EfficientNetB0, named GECM-EfficientNet. The network structure is shown in Figure 4. First, the number of MBConv modules is adjusted, which reduces the model parameters. Secondly, we use the effiicient channel attention (ECA) module [45] to replace the SE module, which solves the shortcomings of the dimensionality reduction operation. Subsequently, parallel connections are made between the coordinate attention (CA) module [46] and the ECA module, enabling spatial weighting operation. Finally, transfer learning is used to initialize the model parameters during training.

#### 3.4.1. Optimising the Network Structure

EfficientNetB0 repeatedly stacks the MBConv module to obtain excellent feature-extraction capabilities. As the network deepens, more channels in the convolutional layer are used to generate more detailed filters, which results in larger parameters. With the structure of the MBConv modules unchanged, the number of MBConv modules was reduced in the deeper network layers, which better balanced the accuracy and parameters of the model. The adapted model was named G-EfficientNet. Table 2 shows a comparison in network structure between EfficientNet and G-EfficientNet.

#### 3.4.2. Efficient Channel Attention

Based on SENet, ECANet proposes the ECA module. ECANet shows that the dimensionality reduction operation in the SE module carries side effects. The ECA module implements a local cross-channel interaction strategy without dimensionality reduction by one-dimensional convolution, effectively improving performance with lower parameters. In this paper, the SE module is replaced by an ECA module, which avoids the side effects of the dimensionality reduction operation and with fewer parameters.

**Figure 5:**
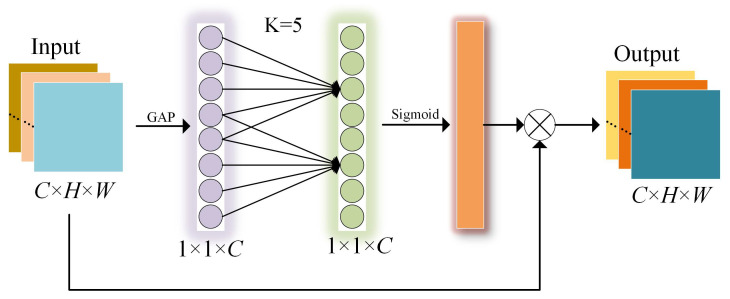
Efficient channel attention module.

Figure 5 shows the working process of the ECA module. First, to obtain the global features without dimensionality reduction, the global average pooling (GAP) is performed on the input feature map. Then, channel weights are generated by one-dimensional convolution and sigmoid function. Finally, the channel weights and the input feature map are multiplied channel by channel, which implements the channel weighting operation.

In the operation of GAP, the input feature map is compressed by global average pooling. After completing GAP, we can obtain global features of dimension 1×1×C. Equation (1) shows the calculation process:

(1) $$z_{c}=\frac{1}{H\times W}\sum_{i=1}^{H}\sum_{j=1}^{W}u_{c}\left(i,j\right),z\in R^{c}.$$

In Equation (3), $z_{c}$ denotes the output features on each channel that uses global average pools. *H* and *W* denote the height and width of the input feature map. *i* and *j* denote the feature value coordinates of the input feature map.

After implementing GAP, the cross-channel interaction strategy is implemented by one-dimensional convolution with size *k*. The parameter *k* is generated through an adaptive function, representing the coverage of the local cross-channel interaction. Equation (2) demonstrates the calculation principle. In Equation (2), *C* is the total number of channels, and |x| odd denotes the nearest odd number to *x*. Finally, channel weights are generated by sigmoid and multiplied with the input feature map by channel. We have

(2) $$k=\varphi \left(C\right)={\left|\frac{\log_{2}C+1}{2}\right|}_{odd}.$$

#### 3.4.3. Coordinate Attention

**Figure 6:**
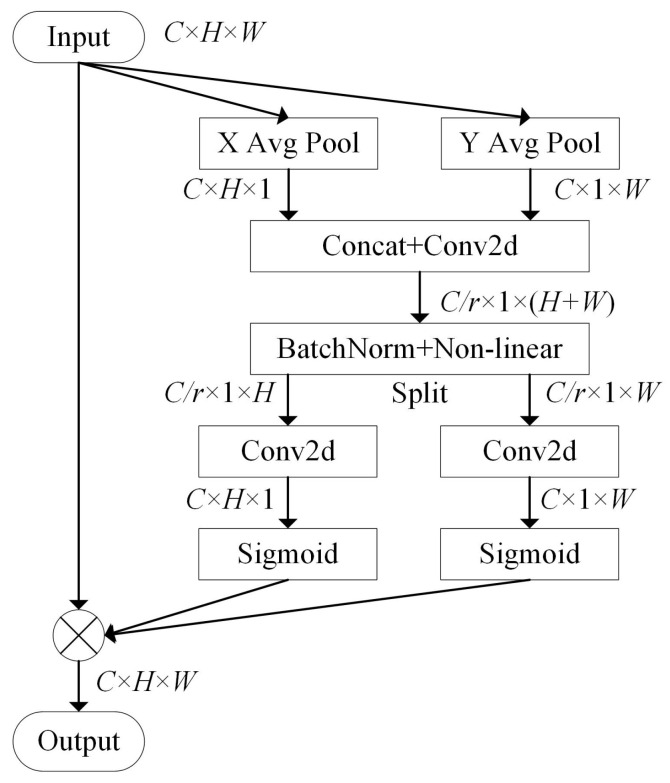
Coordinate attention module.

The ECA module only considers channel weight assignment, which ignores the feature map’s spatial information. CA module embeds location information into the channel weighting operation, allowing the feature map to be weighted by spatial and channel information. Figure 6 shows the structure of CA module. In this paper, we connect the CA module and the ECA module in parallel not only to calibrate the feature maps based on channel information, but also to introduce spatial information.

First, the CA module implements location information embedding for the input feature map X. The dimension of X is H×W×C. Average pooling is used for each channel along the horizontal and vertical directions, with pooling kernels the size of (*H*,1) and (1,*W*). In the *c*th channel, Equations (3) and (4) show the output of the *h*th row and *w*th column:

(3) $$z_{c}^{h}\left(h\right)=\frac{1}{W}\sum_{0\leq i<W}x_{c}\left(h,i\right)$$

(4) $$z_{c}^{w}\left(w\right)=\frac{1}{H}\sum_{0\leq j<H}x_{c}\left(j,w\right).$$

The components of the input feature map are $x_{c}$(*h*,*i*) and $x_{c}$(*j*,*w*), and the coordinates of the components are (*h*,*i*) and (*j*,*w*), and the channel is *c*. $z^{h}$ and $z^{w}$ denote the average pooled output along the horizontal and vertical directions. $z_{c}^{h}$(*h*) and $z_{c}^{w}$(*w*) denote the output component of the *c*th channel in row *h* and column *w*.

Equation (5) shows the next steps. First, the feature map is concatenated, obtained by the pooling operation. Next, the channels are compressed through a standard convolution $F_{1}$ with 1×1. Finally, the intermediate output *m* is obtained through a nonlinear activation layer δ, choosing h-swish as the activation function:

(5) $$m=\delta (F_{1}\left(\left[z^{h},z^{w}\right]\right)).$$

Then, the intermediate output *m* is sliced into two feature maps along the spatial dimension. The feature maps are represented as $m^{h}$ and $m^{w}$, and two 1×1 standard convolutions $F_{h}$ and $F_{w}$ transform them to the same channels with the input feature map X. The activation is then performed by the sigmoid function (σ). The calculation is shown in Equations (6) and (7):

(6) $$g^{h}=\sigma \left(F_{h}\left(m^{h}\right)\right)$$

(7) $$g^{w}=\sigma \left(F_{w}\left(m^{w}\right)\right).$$

In the above equation, $g^{h}$ and $g^{w}$ represent the coordinate attention weights along the horizontal and vertical directions. The final output of the CA module is shown in Equation (8), where $x_{c}$(*i*,*j*) and $y_{c}$(*i*,*j*) correspond to the values in the input and output feature maps with the coordinate (*i*,*j*) and the channel *c*:

(8) $$y_{c}\left(i,j\right)=x_{c}\left(i,j\right)\times g_{c}^{h}\left(i\right)\times g_{c}^{w}\left(j\right).$$

#### 3.4.4. Transfer Learning

Transfer learning [47,48] allows knowledge learned in different domains or tasks to be transferred, which can reduce training time and improve performance. In transfer learning, domain *D* is the subject of learning. The domain is divided into the source domain $D_{s}$ and the target domain $D_{t}$. They consist of the data *X* and the probability distribution *P*(*X*) that generates *X*, which can be expressed as $D=\left\{X,P(X)\right\}$. Task *T* is the goal of learning, divided into the source task $T_{s}$ and the target task $T_{t}$. The task consists of the label space *Y* and the prediction function *f*(·), which can be expressed as $T=\left\{Y,f(\cdot )\right\}$.

Given the source domain $D_{s}$ and the source task $T_{s}$, the target domain $D_{t}$ and the target task $T_{t}$. With $D_{s}\neq D_{t}$ or $T_{s}\neq T_{t}$, transfer learning solves the target task $T_{t}$ in the target domain $D_{t}$, through knowledge learned in the source domain $D_{s}$ and the source task $T_{s}$. This paper implements transfer learning, through the weights of EfficientNetB0 on the ImageNet dataset.

### 3.5. Experimental Settings

During the training, the process is accelerated by the Tesla P100. The dataset is divided into the training dataset and the test dataset according to 8:2. During model training, the generalization capability of the model is enhanced by data augmentation, with such measures as random size cropping, flipping, and luminance transformations. Adam was chosen as the model optimizer. The learning rate prevented overfitting with cosine annealing [49], the initial learning rate was 0.001, and the cosine annealing parameter was 0.01. The loss function was chosen as cross-entropy. The training period is 200, and one batch is trained with 16 images. This paper sets up the following experiments for analysis and discussion.

(1) Ablation experiments of the improved model, verifying each improvement’s contribution to the model performance.
(2) Comparison experiments between the improved model and the mainstream model. All models were trained and tested on both the self-built dataset and the TrashNet dataset, verifying the level of advancement of the improved models.
(3) Model classification accuracy and inference time test. The model was deployed on a Raspberry Pi 4B for testing, verifying the accuracy and real-time performance of the model.

## 4. Results and Discussion

### 4.1. Ablation Experiments

**Figure 7:**
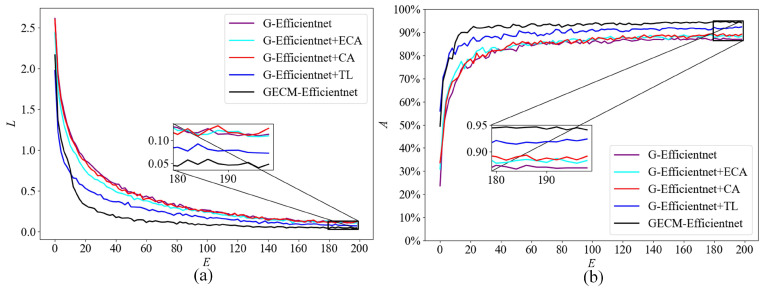
The results of the ablation experiment on the self-built dataset. Subfigure (**a**) shows the loss curve, where the loss value is calculated on the training dataset. Subfigure (**b**) shows the accuracy curve, where the accuracy is calculated on the test dataset.

To validate the contribution of each improvement. We selected the top accuracy and parameters as metrics, and experiments were conducted on the self-built dataset. In order to demonstrate that the network structure can be optimized by streamlining the number of MBConv modules, we compare G-EfficientNet with EfficientNetB0. To prove that the ECA module is lighter and more efficient, the SE module was replaced with an ECA module for the experiments. To prove that the CA module can introduce spatial information, the SE and CA modules are connected in parallel. To prove that transfer learning can optimize the model parameters. We initialized the model parameters by EfficientNetB0 weights, trained on the ImageNet dataset. Figure 7 shows the training loss and test accuracy curves of the model. *L* is the training loss, which is calculated on the training dataset. *A* is the test accuracy, which is calculated on the test dataset. *E* is training epoch. Obviously, GECM-EfficientNet achieves the best test accuracy and converges quickly.

Table 3 shows the performance parameters of the above model. *A* is the top1 accuracy of the model, and *P* represents the number of parameters of the mode. As can be seen, the parameters of G-EfficientNet are reduced by 72.2% compared to EfficientNetB0, but the accuracy is only reduced by 1.27%. The SE module is replaced with the ECA module, which reduces parameters by 0.1 M and improves accuracy by 1.48%. There is a parallel connection of the CA module and the SE module, with only 0.2 M additional parameters, but with a 1.80% increase in accuracy. Optimizing the model parameters by transfer learning, the parameters remain the same with a 4.53% improvement in accuracy.

First, the number of MBConv modules in EfficientNetB0 is adjusted, which acquires the lighter model G-EfficientNet, Next, we improve the MBConv module. We replaced the SE module with the ECA module, connecting the CA module in parallel. During model training, the model parameters are initialized by transfer learning. Ultimately, GECM-EfficientNet was designed. Compared to EfficientNetB0, the model accuracy was improved by 5.7% on the self-built dataset, with 69.73% fewer parameters.

### 4.2. Comparison and Analysis of Models

To verify the level of advancement of the improved model, GECM-EfficientNet was compared with the mainstream model, with experiments completed on both the self-built dataset and TrashNet dataset. Finally, the models were deployed on the Raspberry Pi 4B for classification and inference time test. We selected lightweight models such as EfficientNetB0, MobileNetv2, MobileNetv3 [50], and ShuffleNetv2, and large models such as GoogleNet, DenseNet121, ResNet50, Inceptionv3, and VGG16.

#### 4.2.1. On the Self-Built Dataset

**Figure 8:**
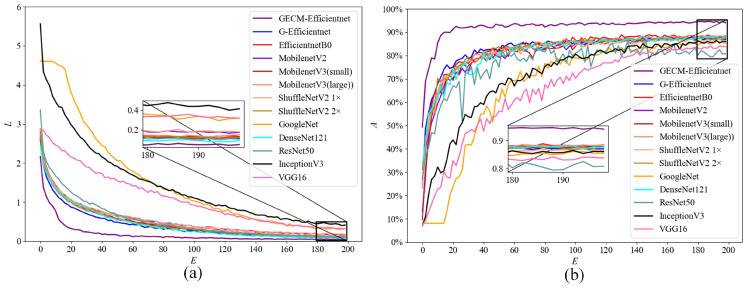
The results of the model performance comparison on the self-built dataset. Subfigure (**a**) shows the loss curve, where the loss value is calculated on the training dataset. Subfigure (**b**) shows the accuracy curve, where the accuracy is calculated on the test dataset.

Figure 8 shows the training loss and test accuracy curves on the self-built dataset. As can be seen, GECM-EfficientNet is in the lead, achieving an accuracy of approximately 90% in only 20 epochs.

The test accuracy, parameters, and single inference time of models are shown in Table 4, where *T* represents the single inference time of model on the Raspberry Pi 4B. As can be seen, the lightweight model achieves similar or higher accuracy than the large network, with fewer parameters and high real-time performance. Among the mainstream models, EfficientNetB0 achieved the highest accuracy (88.81%) and low parameters (4.03 M), with single inference taking 0.2 s, second only to MobileNetv3, ShuffleNetv2 1×, and MobileNetv2. Our proposed GECM-EfficientNet, with only 1.23 M parameters, achieves 94.54% accuracy with a single inference time of 146 ms. The reasons for this analysis are as follows: (1) We adapt the number of MBConv modules to obtain G-EfficientNet, which is a lightweight and excellent model. (2) The ECA module is lighter than the SE module, eliminating dimensionality reduction operations’ side effects. (3) Embedding of location information through the CA attention module, which allows for spatially weighted operations. (4) Optimization of model parameters through EfficientNetB0 weights on the ImageNet dataset, which speeds up convergence and improves accuracy.

#### 4.2.2. On the Trashnet Dataset

**Figure 9:**
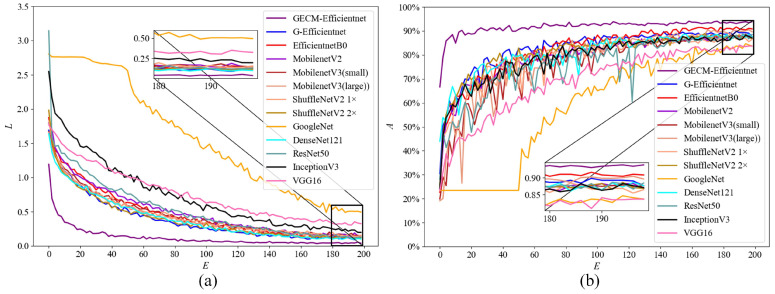
The results of the model performance comparison on the TrashNet dataset. Subfigure (**a**) shows the loss curve, where the loss value is calculated on the training dataset. Subfigure (**b**) shows the accuracy curve, where the accuracy is calculated on the test dataset.

To further validate the advances of the improved model, comparison experiments were set up on the TrashNet dataset. Figure 9 shows the training loss and test accuracy curves of models. It is evident that GECM-EfficientNet achieves the highest accuracy and converges quickly.

The experimental results are shown in Table 5. Among the mainstream models, the proposed GECM-EfficientNet is in the leading position. It achieved the highest accuracy (94.23%) and the lowest parameters (1.23 M). As can be verified, GECM-EfficientNet is a lightweight and excellent model. In order to further validate the superiority of the improved model, GECM-EfficientNet was compared with other related studies. Among them, the literature [4] proposes RecycleNet with an accuracy of 81%. Ref. [51] implements a dual fusion approach by PSO and GA, which achieves 94.11% and 94.58% accuracy. Among these related studies, GECM-EfficientNet is also in the lead, with an accuracy similar to the literature [10], the literature [11], and the GA (Ahmad et al. [51]). However, they focus on improving the accuracy of large models, but with large parameters and poor real-time performance. The GECM-EfficientNet parameters are few, but achieve high accuracy. On resource-constrained edge devices, GECM-EfficientNet offers good prospects for application.

### 4.3. System Testing

#### 4.3.1. Speed Test

**Figure 10:**
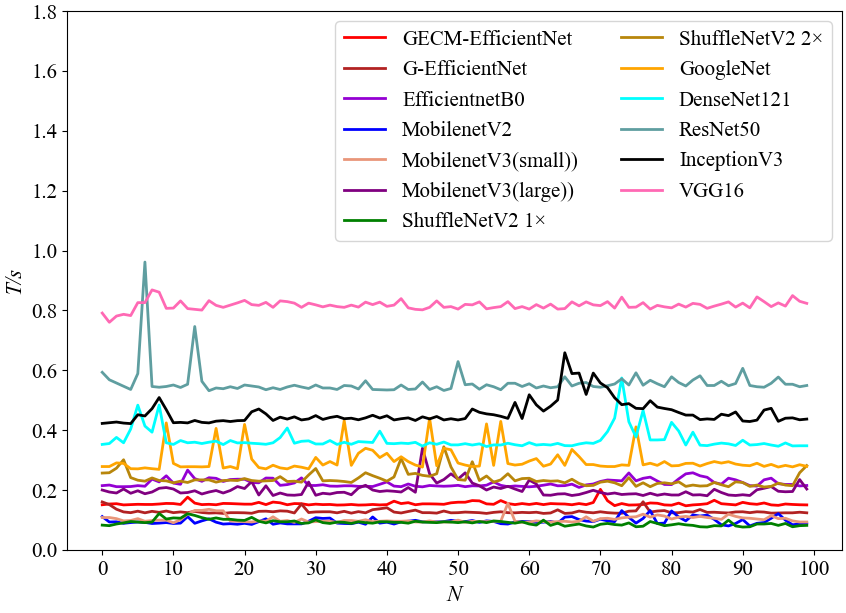
Inference time on the Raspberry Pi 4B.

In order to verify the real-time performance of GECM-EfficientNet, the above model was deployed to the Raspberry Pi 4B for testing. Figure 10 shows the single inference time. *N* is the number of inferences. T/s represents the single inference time, which is in seconds. Among the mainstream models, GECM-EfficientNet has a significant advantage on real-time performance. The average single inference time of GECM-EfficientNet is 146 ms, meeting the real-time performance requirements of waste classification.

#### 4.3.2. Classification Test

**Figure 11:**
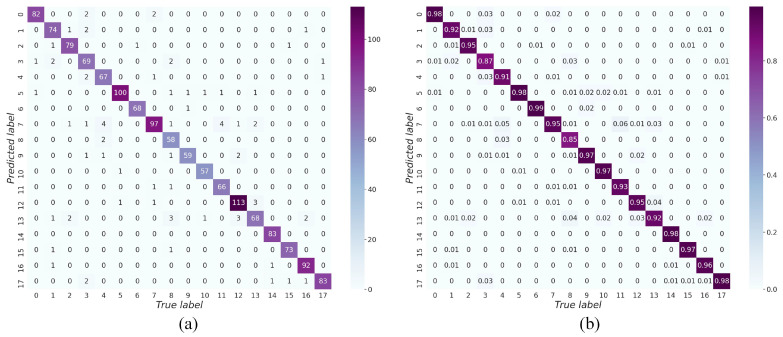
Confusion matrix of GECM-EfficientNet on the test dataset of the self-built dataset. The x-axis and y-axis represent the true and predicted categories of waste respectively, and the different categories are represented by numbers. Subfigure (**a**) shows the number of predictions for each waste category. Subfigure (**b**) is obtained by normalizing subfigure (**a**).

The confusion matrix is plotted through the test dataset of the self-built dataset, which shows the prediction results for the different categories. Figure 11 shows the results. The rows and columns of the matrix indicate the true and predicted values for waste categories. The values on the diagonal in Figure 11a indicate the number of correctly sorted waste items, whereas the values outside the diagonal indicate the number of incorrectly sorted waste items. Figure 11a is normalized to give Figure 11b, the diagonal values indicate the accuracy of the classification. As can be seen, single category accuracy remains mostly above 90% and up to 99%. Category 8 (waste dry batteries) is the least accurate (85%) because the waste dry battery’s small, cylindrical shape resembles a cigarette butt. The confusion matrix shows that the GECM-EfficientNet can accurately distinguish between different categories of waste.

**Figure 12:**
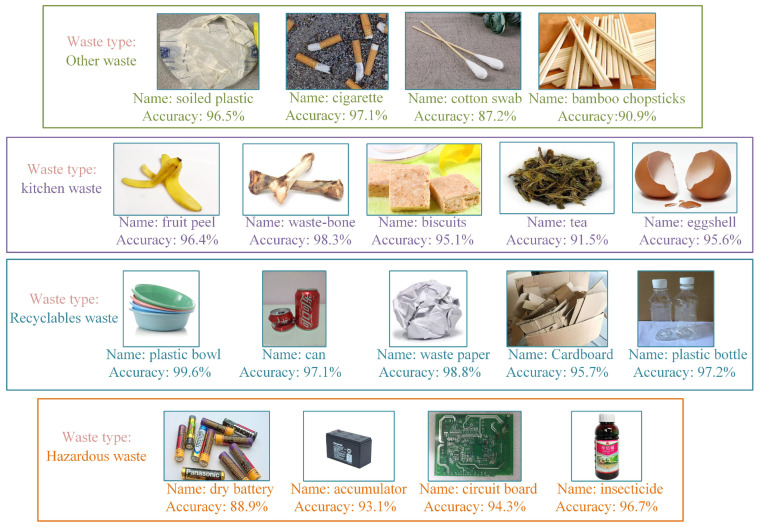
Test results for partial waste images, where the waste type, name and classification accuracy are shown.

Selected waste images from the test dataset for testing. Figure 12 shows the category and accuracy of the waste tested. The four types of waste are framed by different colors. The waste type indicates the type of waste containing other waste, kitchen waste, recyclable waste, and hazardous waste. The name indicates the category of waste and accuracy indicates the classification accuracy, which is expressed as a percentage. As can be seen, the model can achieve an accurate classification of different waste. It can be proven that the GECM-EfficientNet has a high classification accuracy, which satisfies the requirements of waste-sorting devices.

### 4.4. Discussion of Intelligent Waste Bin

Most of the existing normal waste recycling devices are set up with different bins, which then wait for residents to sort and put out waste manually. However, residents often do not have enough knowledge of sorting, and there is difficulty in translating willingness into action. As a result, this paper designs an intelligent waste bin that automatically sorts and recycles household waste. The intelligent waste bin costs just 2500 CNY. The waste-classification model is trained with 18 categories of waste images. However, for other categories of waste, if the model is trained with enough images of the waste, it can also correctly identify the category of waste. The intelligent waste bin recycles waste to different bins through the combined movement of the paddle plate and baffle plate. This mechanical structure can effectively recycle solid waste, but it may not be pleasant for liquid waste, which may be partially left behind. Therefore, the recycling of liquid waste will be one of future highlights.

Nowadays, some academics are also researching intelligent waste-sorting devices. Ref. [9] designed a smart waste bin based on ResNet34, which can dichotomize waste with a single inference time of 950 ms. Ref. [52] constructed a smart bin based on inceptionv3, which can recycle waste into two bins. These smart devices are based on large models for waste classification, which has significance for automatic waste sorting and recycling. However, although they achieve high accuracy, they perform weakly in real time, which can cause unhappiness for users. Ref. [15] build an intelligent waste-sorting system through a lightweight model (MobileNetv3), in which the waste classification model is deployed in the cloud. Cloud deployment avoids the expense of local computing resources, but this is heavily dependent on the Internet. The highlight in this paper is that a lightweight waste-classification model is proposed, which is directly deployed on the embedded device, avoiding Internet dependencies. The model is high in real-time accuracy, with a single inference time of 146 ms. At the same time, this paper proposes the intelligent waste bin that can sort waste more carefully, and the waste will be recycled into four bins. It can be placed in airports, schools, and shopping malls, which contributes to environmental protection and resource recycling.

## 5. Conclusions

With the increasing focus on environmental safety and resource recycling, society is calling on residents to sort their waste. This requires residents to be knowledgeable about the different categories of waste, which makes it very difficult to sort waste. For this, intelligent waste-sorting devices would be an effective solution. This paper introduces computer vision technology to waste classification, proposing a lightweight and efficient waste-classification model (GECM-EfficientNet), and an intelligent waste bin is designed based on GECM-EfficientNet. On the self-built household waste dataset, GECM-EfficientNet achieved the accuracy of 94.54%, with a single inference time of 146 ms. The intelligent waste bin enables the automatic sorting and recycling of waste, improving the efficiency of waste sorting. It is relevant for environmental protection and resource recycling, but also beneficial for the country’s sustainable development. The main work and contributions in this paper are as follows.

(1) We chose the lightweight EfficientNetB0 as the baseline model. The MBConv module is first streamlined, optimizing the model structure and reducing complexity. Then, the ECA module and CA module are connected in parallel, replacing the SE module in the MBConv module, which implements the feature map’s spatial and channel weighting operations.
(2) In the training strategy, the model parameters are initialized by transfer learning, which improves the model performance and convergence speed.
(3) We verify the superiority of the GECM-EfficientNet performance with the self-built dataset and the TrashNet dataset. Among the many mainstream models and related research, GECM-EfficientNet is in the lead, with outstanding performance in accuracy and real-time performance.
(4) We design an intelligent waste bin and implement waste classification through GECM-EfficientNet.The model first identifies the input waste, then sorts and recycles into the corresponding bins by the execution structure. This provides a new solution for alleviating the environmental crisis and achieving a circular economy.

In waste sorting and recycling, some research results have been made in this paper, but the following limitations still exist. (1) Waste-classification models can only identify 18 categories of household waste. In reality, there are many categories of waste, and the dataset needs to be expanded later. (2) Consider the use of semi-supervised learning, which makes use of the vast amount of unlabelled image data for learning, facilitating the performance of the classification model. (3) The current mechanical structure is unable to recycle mixed waste. In future work, it may be effective to design a loading device that can separate mixed waste to single waste. In addition, image segmentation and object-detection techniques can identify the different components of mixed waste.

## Figures and Tables

**Table 1:** Self-built household waste dataset.

Category	Name	Number	Category	Name	Number
Kitchen waste	Fruit flee	425	Recyclable waste	Plastic bowl	417
	Waste bone	379		Can	374
	Biscuits	484		Waste paper	510
	Tea	426		Cardboard	348
	Eggshell	401		Plastic bottle	511
Other waste	Soiled plastic	422	Hazardous waste	Dry battery	342
	Cigarette	395		Accumulator	307
	Cotton swab	595		Circuit board	299
	Chopsticks	371		Insecticide	355

**Table 2:** G-EfficientNet model structure.

Stage	Operator	Resolution	Channel	Repeats (Orignal)	Repeats (Adapt)
1	Conv3×3	224×224	32	1	1
2	MBConv1, k3×3	112×112	16	1	1
3	MBConv6, k3×3	112×112	24	2	2
4	MBConv6, k5×5	56×56	40	2	2
5	MBConv6, k3×3	28×28	80	3	2
6	MBConv6, k5×5	14×14	112	3	1
7	MBConv6, k5×5	14×14	192	4	0
8	MBConv6, k3×3	7×7	320	1	1
9	Conv1×1&Pooling&FC	7×7	1280	1	1

**Table 3:** Ablation experiments on the self-built dataset.

Model	ECA Module	CA Module	Transfer Learning	*A*/%	*P*/M
EfficientNetB0	-	-	-	88.81	4.03
G-EfficientNet	-	-	-	87.54	1.12
	✓	-	-	89.02	1.02
	-	✓	-	89.35	1.32
	-	-	✓	91.76	1.12
	✓	✓	✓	94.54	1.22

**Table 4:** Results of experiments with the self-built dataset.

Model	*A*/%	*P*/M	*T*/ms	Model	*A*/%	*P*/M	*T*/ms
GECM-EfficienNet	94.54	1.23	146.82	ShuffleNetv2 1×	86.73	1.27	101.51
G-EfficientNet	87.54	1.12	127.78	ShuffleNetv2 2×	88.13	5.38	225.42
EfficientNetB0	88.81	4.03	211.21	GoogleNet	85.92	5.61	278.73
MobileNetv2	87.72	2.25	93.26	DenseNet121	88.02	6.97	367.51
MobileNetv3 (small)	87.75	1.54	95.89	ResNet50	84.7	23.55	524.35
MobileNetv3 (large)	88.47	4.23	207.78	Inceptionv3	86.4	21.82	451.49
VGG16	83.88	134.33	837.95	-	-	-	-

**Table 5:** Experimental results on the TrashNet dataset.

Model	*A*/%	Model	*A*/%	Model	*A*/%
GECM-EfficienNet	94.23	ShuffleNetv2 1×	87.27	VGG16	84.09
G-EfficientNet	89.26	ShuffleNetv2 2×	88.67	[11]	94.02
EfficientNetB0	91.65	GoogleNet	84.69	PSO( Ahmad et al. [51])	94.11
MobileNetv2	88.86	DenseNet121	88.46	[10]	95.87
MobileNetv3 (small)	88.27	ResNet50	88.86	[4]	81
MobileNetv3 (large)	90.45	Inceptionv3	88.27	GA( Ahmad et al. [51])	94.58

## Data Availability

Not applicable.
